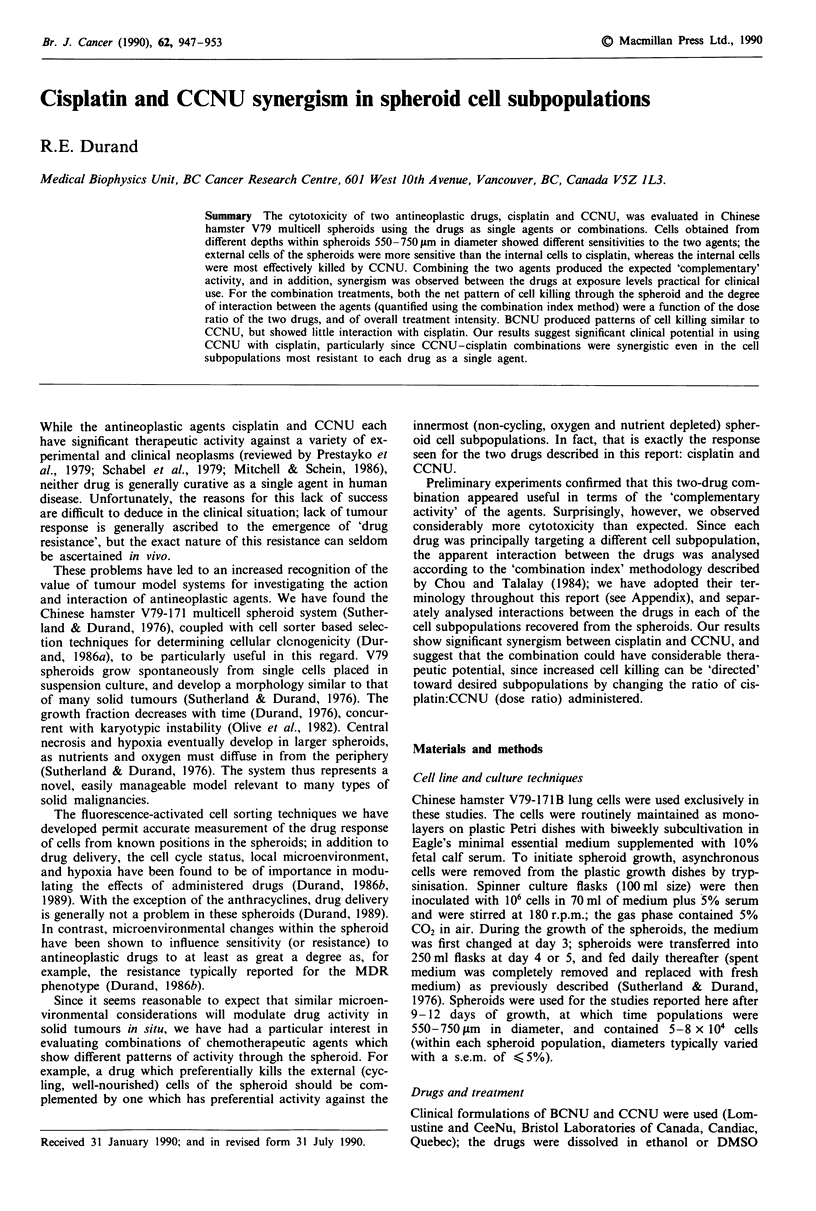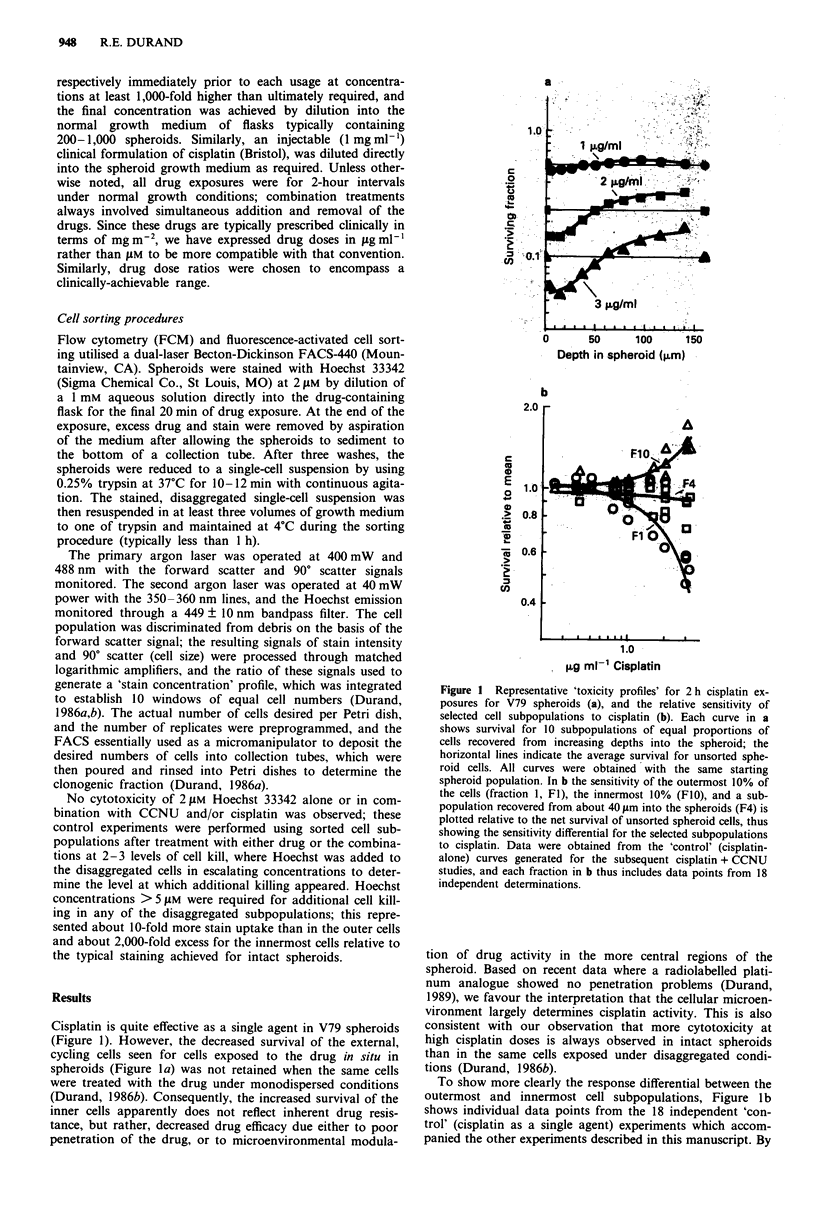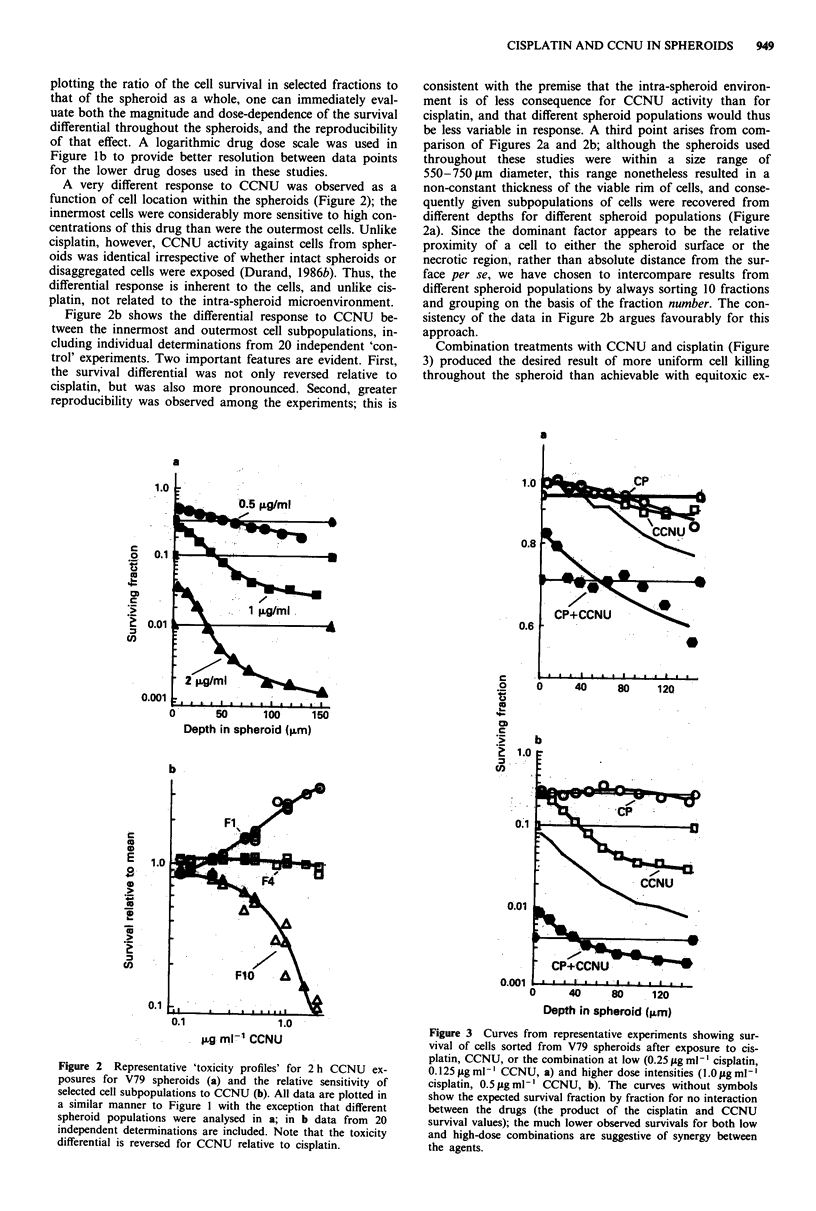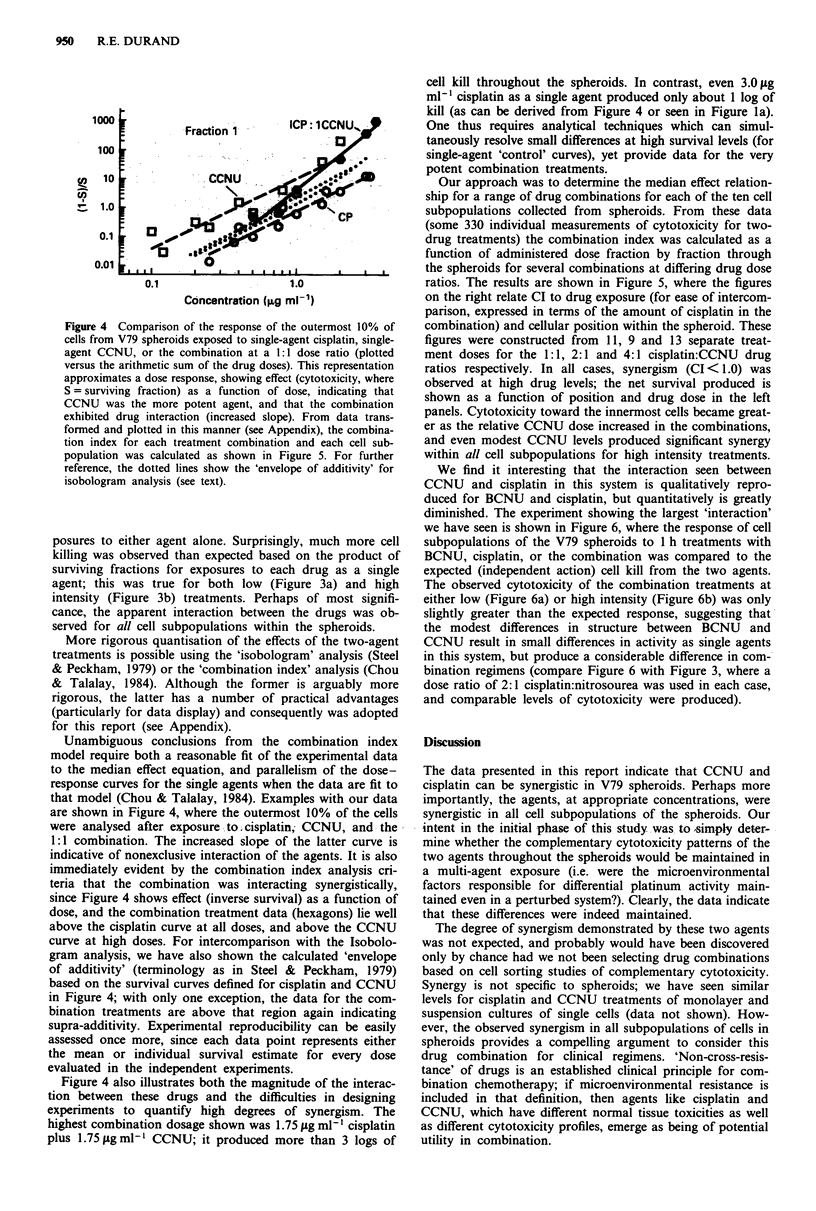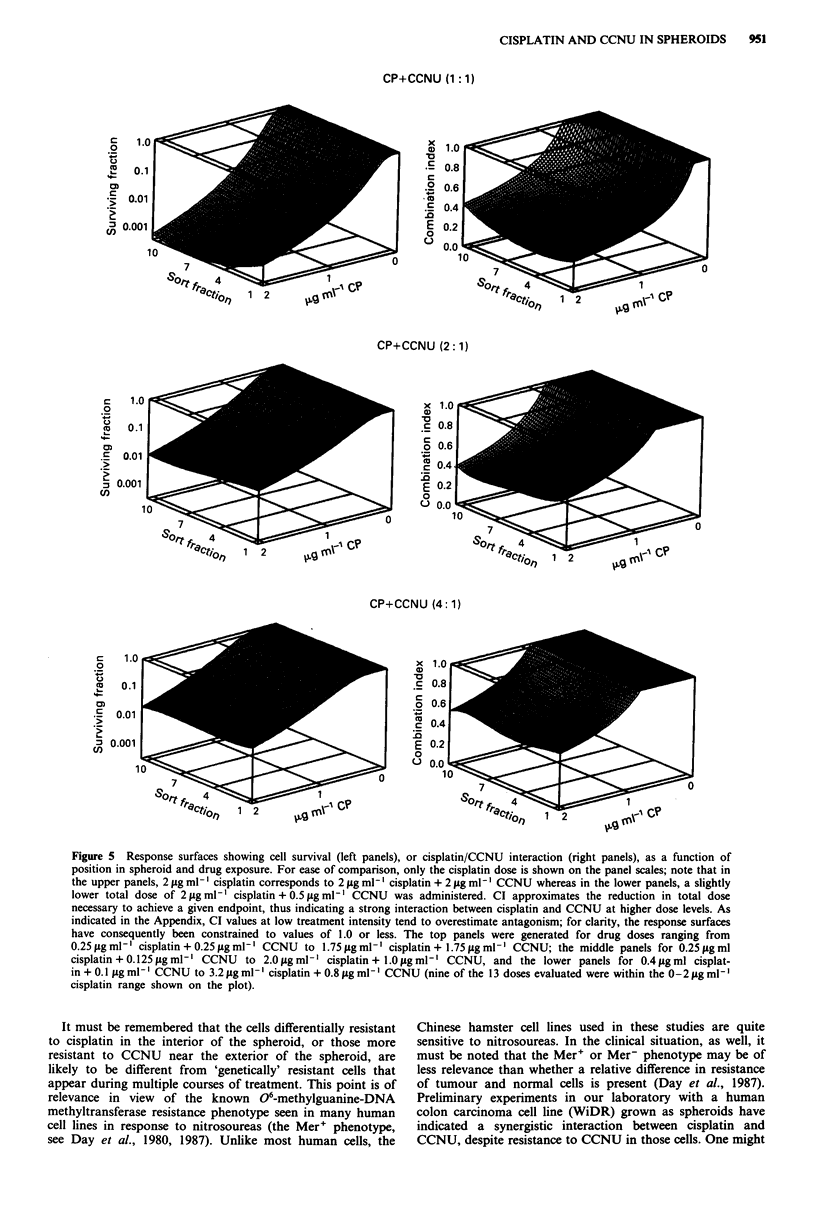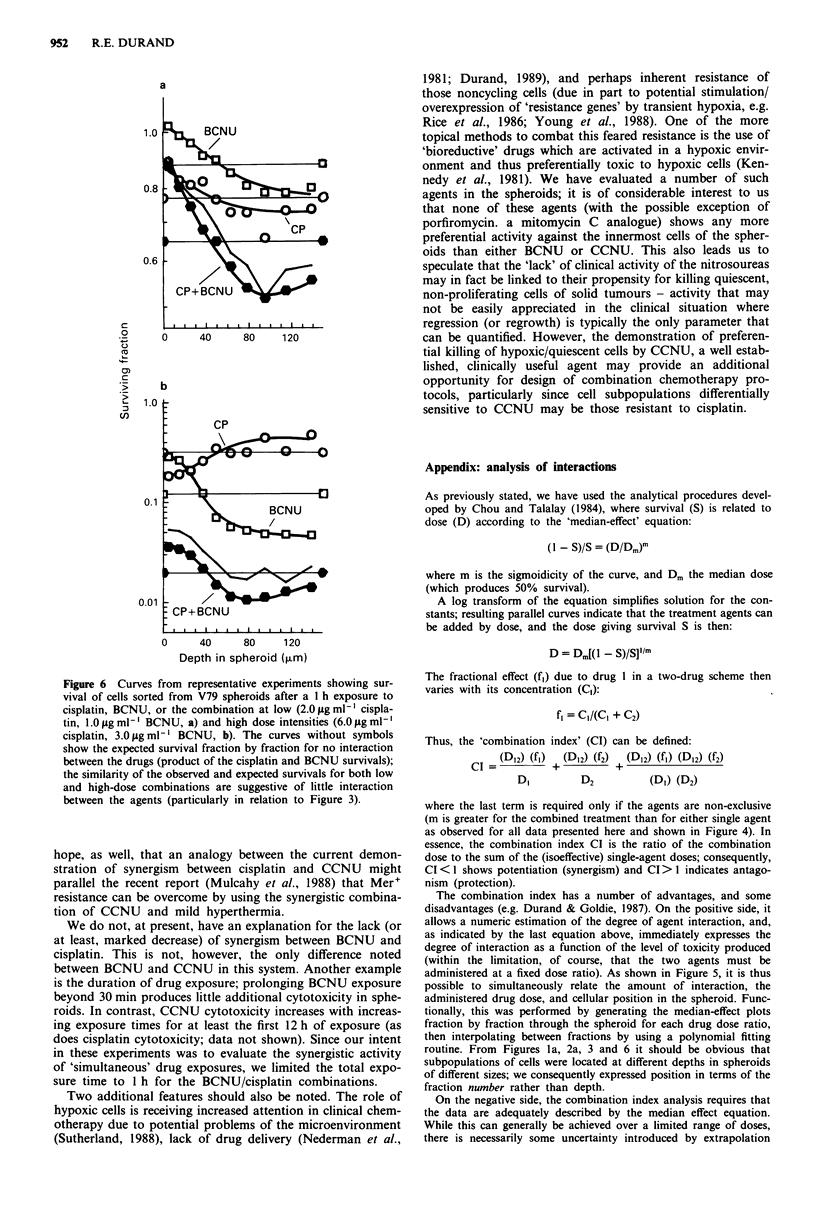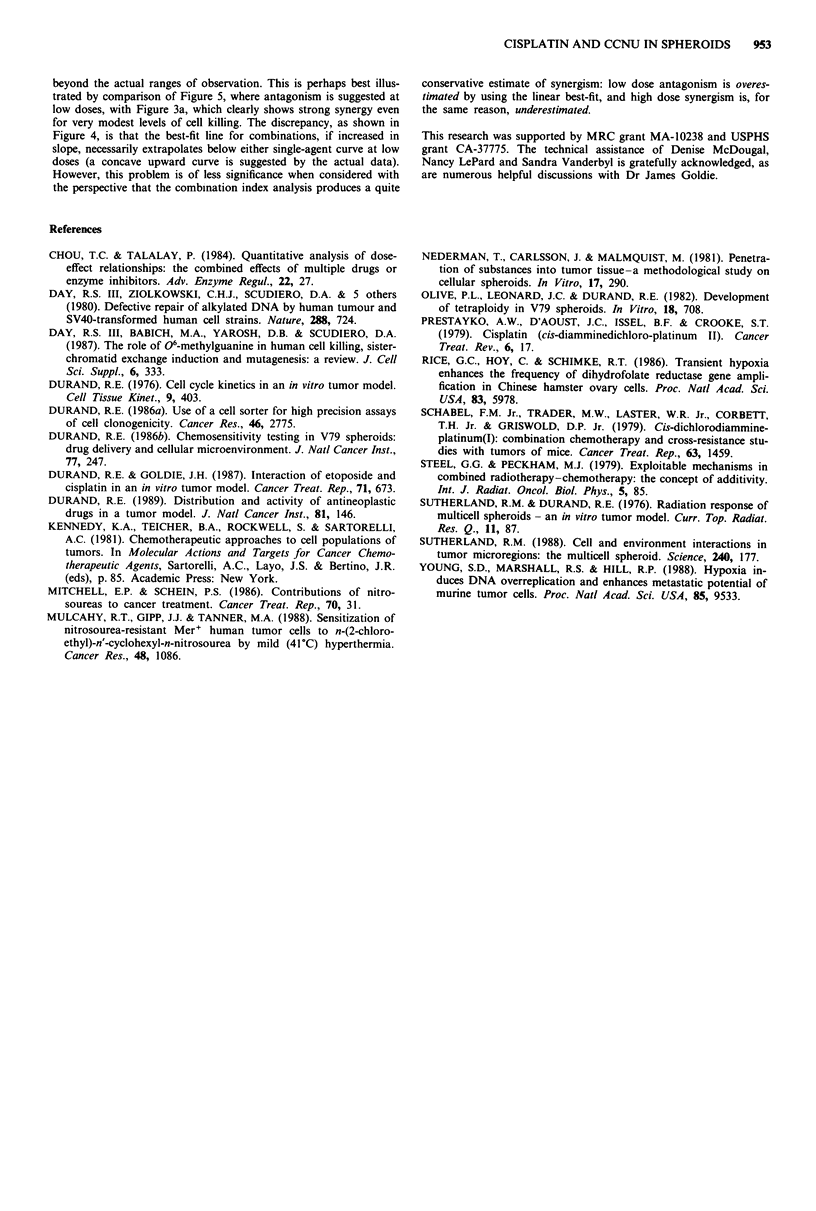# Cisplatin and CCNU synergism in spheroid cell subpopulations.

**DOI:** 10.1038/bjc.1990.415

**Published:** 1990-12

**Authors:** R. E. Durand

**Affiliations:** Medical Biophysics Unit, BC Cancer Research Centre, Vancouver, Canada.

## Abstract

The cytotoxicity of two antineoplastic drugs, cisplatin and CCNU, was evaluated in Chinese hamster V79 multicell spheroids using the drugs as single agents or combinations. Cells obtained from different depths within spheroids 550-750 microns in diameter showed different sensitivities to the two agents; the external cells of the spheroids were more sensitive than the internal cells to cisplatin, whereas the internal cells were most effectively killed by CCNU. Combining the two agents produced the expected 'complementary' activity, and in addition, synergism was observed between the drugs at exposure levels practical for clinical use. For the combination treatments, both the net pattern of cell killing through the spheroid and the degree of interaction between the agents (quantified using the combination index method) were a function of the dose ratio of the two drugs, and of overall treatment intensity. BCNU produced patterns of cell killing similar to CCNU, but showed little interaction with cisplatin. Our results suggest significant clinical potential in using CCNU with cisplatin, particularly since CCNU-cisplatin combinations were synergistic even in the cell subpopulations most resistant to each drug as a single agent.


Br. J. Cancer (1990), 62, 947-953 © Macmillan Press Ltd., 1990

### Cisplatin and CCNU synergism in spheroid cell subpopulations

R.E. Durand

*Medical Biophysics Unit, BC Cancer Research Centre, 601 West 10th Avenue, Vancouver, BC, Canada V5Z 1L3.*

**Summary** The cytotoxicity of two antineoplastic drugs, cisplatin and CCNU, was evaluated in Chinese hamster V79 multicell spheroids using the drugs as single agents or combinations. Cells obtained from different depths within spheroids 550-750 µm in diameter showed different sensitivities to the two agents; the external cells of the spheroids were more sensitive than the internal cells to cisplatin, whereas the internal cells were most effectively killed by CCNU. Combining the two agents produced the expected 'complementary' activity, and in addition, synergism was observed between the drugs at exposure levels practical for clinical use. For the combination treatments, both the net pattern of cell killing through the spheroid and the degree of interaction between the agents (quantified using the combination index method) were a function of the dose ratio of the two drugs, and of overall treatment intensity. BCNU produced patterns of cell killing similar to CCNU, but showed little interaction with cisplatin. Our results suggest significant clinical potential in using CCNU with cisplatin, particularly since CCNU-cisplatin combinations were synergistic even in the cell subpopulations most resistant to each drug as a single agent.

While the antineoplastic agents cisplatin and CCNU each have significant therapeutic activity against a variety of experimental and clinical neoplasms (reviewed by Prestayko *et al.*, 1979; Schabel *et al.*, 1979; Mitchell & Schein, 1986), neither drug is generally curative as a single agent in human disease. Unfortunately, the reasons for this lack of success are difficult to deduce in the clinical situation; lack of tumour response is generally ascribed to the emergence of 'drug resistance', but the exact nature of this resistance can seldom be ascertained *in vivo*.

These problems have led to an increased recognition of the value of tumour model systems for investigating the action and interaction of antineoplastic agents. We have found the Chinese hamster V79-171 multicell spheroid system (Sutherland & Durand, 1976), coupled with cell sorter based selection techniques for determining cellular clonogenicity (Durand, 1986*a*), to be particularly useful in this regard. V79 spheroids grow spontaneously from single cells placed in suspension culture, and develop a morphology similar to that of many solid tumours (Sutherland & Durand, 1976). The growth fraction decreases with time (Durand, 1976), concurrent with karyotypic instability (Olive *et al.*, 1982). Central necrosis and hypoxia eventually develop in larger spheroids, as nutrients and oxygen must diffuse in from the periphery (Sutherland & Durand, 1976). The system thus represents a novel, easily manageable model relevant to many types of solid malignancies.

The fluorescence-activated cell sorting techniques we have developed permit accurate measurement of the drug response of cells from known positions in the spheroids; in addition to drug delivery, the cell cycle status, local microenvironment, and hypoxia have been found to be of importance in modulating the effects of administered drugs (Durand, 1986*b*, 1989). With the exception of the anthracyclines, drug delivery is generally not a problem in these spheroids (Durand, 1989). In contrast, microenvironmental changes within the spheroid have been shown to influence sensitivity (or resistance) to antineoplastic drugs to at least as great a degree as, for example, the resistance typically reported for the MDR phenotype (Durand, 1986*b*).

Since it seems reasonable to expect that similar microenvironmental considerations will modulate drug activity in solid tumours *in situ*, we have had a particular interest in evaluating combinations of chemotherapeutic agents which show different patterns of activity through the spheroid. For example, a drug which preferentially kills the external (cycling, well-nourished) cells of the spheroid should be complemented by one which has preferential activity against the innermost (non-cycling, oxygen and nutrient depleted) spheroid cell subpopulations. In fact, that is exactly the response seen for the two drugs described in this report: cisplatin and CCNU.

Preliminary experiments confirmed that this two-drug combination appeared useful in terms of the 'complementary activity' of the agents. Surprisingly, however, we observed considerably more cytotoxicity than expected. Since each drug was principally targeting a different cell subpopulation, the apparent interaction between the drugs was analysed according to the 'combination index' methodology described by Chou and Talalay (1984); we have adopted their terminology throughout this report (see Appendix), and separately analysed interactions between the drugs in each of the cell subpopulations recovered from the spheroids. Our results show significant synergism between cisplatin and CCNU, and suggest that the combination could have considerable therapeutic potential, since increased cell killing can be 'directed' toward desired subpopulations by changing the ratio of cisplatin:CCNU (dose ratio) administered.

### Materials and methods

### *Cell line and culture techniques*

Chinese hamster V79-171B lung cells were used exclusively in these studies. The cells were routinely maintained as monolayers on plastic Petri dishes with biweekly subcultivation in Eagle's minimal essential medium supplemented with 10% fetal calf serum. To initiate spheroid growth, asynchronous cells were removed from the plastic growth dishes by trypsinisation. Spinner culture flasks (100 ml size) were then inoculated with $10^6$ cells in 70 ml of medium plus 5% serum and were stirred at 180 r.p.m.; the gas phase contained 5% $CO_2$ in air. During the growth of the spheroids, the medium was first changed at day 3; spheroids were transferred into 250 ml flasks at day 4 or 5, and fed daily thereafter (spent medium was completely removed and replaced with fresh medium) as previously described (Sutherland & Durand, 1976). Spheroids were used for the studies reported here after 9-12 days of growth, at which time populations were 550-750 µm in diameter, and contained $5-8 \times 10^4$ cells (within each spheroid population, diameters typically varied with a s.e.m. of $\leqslant 5\%$).

### *Drugs and treatment*

Clinical formulations of BCNU and CCNU were used (Lomustine and CeeNu, Bristol Laboratories of Canada, Candiac, Quebec); the drugs were dissolved in ethanol or DMSO

Received 31 January 1990; and in revised form 31 July 1990.

respectively immediately prior to each usage at concentrations at least 1,000-fold higher than ultimately required, and the final concentration was achieved by dilution into the normal growth medium of flasks typically containing 200–1,000 spheroids. Similarly, an injectable (1 mg ml$^{-1}$) clinical formulation of cisplatin (Bristol), was diluted directly into the spheroid growth medium as required. Unless otherwise noted, all drug exposures were for 2-hour intervals under normal growth conditions; combination treatments always involved simultaneous addition and removal of the drugs. Since these drugs are typically prescribed clinically in terms of mg m$^{-2}$, we have expressed drug doses in $\mu$g ml$^{-1}$ rather than $\mu$M to be more compatible with that convention. Similarly, drug dose ratios were chosen to encompass a clinically-achievable range.

*Cell sorting procedures*

Flow cytometry (FCM) and fluorescence-activated cell sorting utilised a dual-laser Becton-Dickinson FACS-440 (Mountainview, CA). Spheroids were stained with Hoechst 33342 (Sigma Chemical Co., St Louis, MO) at 2 $\mu$M by dilution of a 1 mM aqueous solution directly into the drug-containing flask for the final 20 min of drug exposure. At the end of the exposure, excess drug and stain were removed by aspiration of the medium after allowing the spheroids to sediment to the bottom of a collection tube. After three washes, the spheroids were reduced to a single-cell suspension by using 0.25% trypsin at 37°C for 10–12 min with continuous agitation. The stained, disaggregated single-cell suspension was then resuspended in at least three volumes of growth medium to one of trypsin and maintained at 4°C during the sorting procedure (typically less than 1 h).

The primary argon laser was operated at 400 mW and 488 nm with the forward scatter and 90° scatter signals monitored. The second argon laser was operated at 40 mW power with the 350–360 nm lines, and the Hoechst emission monitored through a 449 ± 10 nm bandpass filter. The cell population was discriminated from debris on the basis of the forward scatter signal; the resulting signals of stain intensity and 90° scatter (cell size) were processed through matched logarithmic amplifiers, and the ratio of these signals used to generate a 'stain concentration' profile, which was integrated to establish 10 windows of equal cell numbers (Durand, 1986*a*,*b*). The actual number of cells desired per Petri dish, and the number of replicates were preprogrammed, and the FACS essentially used as a micromanipulator to deposit the desired numbers of cells into collection tubes, which were then poured and rinsed into Petri dishes to determine the clonogenic fraction (Durand, 1986*a*).

No cytotoxicity of 2 $\mu$M Hoechst 33342 alone or in combination with CCNU and/or cisplatin was observed; these control experiments were performed using sorted cell subpopulations after treatment with either drug or the combinations at 2–3 levels of cell kill, where Hoechst was added to the disaggregated cells in escalating concentrations to determine the level at which additional killing appeared. Hoechst concentrations $> 5\ \mu$M were required for additional cell killing in any of the disaggregated subpopulations; this represented about 10-fold more stain uptake than in the outer cells and about 2,000-fold excess for the innermost cells relative to the typical staining achieved for intact spheroids.

**Figure 1** Representative 'toxicity profiles' for 2 h cisplatin exposures for V79 spheroids (**a**), and the relative sensitivity of selected cell subpopulations to cisplatin (**b**). Each curve in **a** shows survival for 10 subpopulations of equal proportions of cells recovered from increasing depths into the spheroid; the horizontal lines indicate the average survival for unsorted spheroid cells. All curves were obtained with the same starting spheroid population. In **b** the sensitivity of the outermost 10% of the cells (fraction 1, F1), the innermost 10% (F10), and a subpopulation recovered from about 40 $\mu$m into the spheroids (F4) is plotted relative to the net survival of unsorted spheroid cells, thus showing the sensitivity differential for the selected subpopulations to cisplatin. Data were obtained from the 'control' (cisplatin-alone) curves generated for the subsequent cisplatin + CCNU studies, and each fraction in **b** thus includes data points from 18 independent determinations.

### Results

Cisplatin is quite effective as a single agent in V79 spheroids (Figure 1). However, the decreased survival of the external, cycling cells seen for cells exposed to the drug *in situ* in spheroids (Figure 1*a*) was not retained when the same cells were treated with the drug under monodispersed conditions (Durand, 1986*b*). Consequently, the increased survival of the inner cells apparently does not reflect inherent drug resistance, but rather, decreased drug efficacy due either to poor penetration of the drug, or to microenvironmental modulation of drug activity in the more central regions of the spheroid. Based on recent data where a radiolabelled platinum analogue showed no penetration problems (Durand, 1989), we favour the interpretation that the cellular microenvironment largely determines cisplatin activity. This is also consistent with our observation that more cytotoxicity at high cisplatin doses is always observed in intact spheroids than in the same cells exposed under disaggregated conditions (Durand, 1986*b*).

To show more clearly the response differential between the outermost and innermost cell subpopulations, Figure 1b shows individual data points from the 18 independent 'control' (cisplatin as a single agent) experiments which accompanied the other experiments described in this manuscript. By plotting the ratio of the cell survival in selected fractions to that of the spheroid as a whole, one can immediately evaluate both the magnitude and dose-dependence of the survival differential throughout the spheroids, and the reproducibility of that effect. A logarithmic drug dose scale was used in Figure 1b to provide better resolution between data points for the lower drug doses used in these studies.

A very different response to CCNU was observed as a function of cell location within the spheroids (Figure 2); the innermost cells were considerably more sensitive to high concentrations of this drug than were the outermost cells. Unlike cisplatin, however, CCNU activity against cells from spheroids was identical irrespective of whether intact spheroids or disaggregated cells were exposed (Durand, 1986*b*). Thus, the differential response is inherent to the cells, and unlike cisplatin, not related to the intra-spheroid microenvironment.

Figure 2b shows the differential response to CCNU between the innermost and outermost cell subpopulations, including individual determinations from 20 independent 'control' experiments. Two important features are evident. First, the survival differential was not only reversed relative to cisplatin, but was also more pronounced. Second, greater reproducibility was observed among the experiments; this is consistent with the premise that the intra-spheroid environment is of less consequence for CCNU activity than for cisplatin, and that different spheroid populations would thus be less variable in response. A third point arises from comparison of Figures 2a and 2b; although the spheroids used throughout these studies were within a size range of 550–750 μm diameter, this range nonetheless resulted in a non-constant thickness of the viable rim of cells, and consequently given subpopulations of cells were recovered from different depths for different spheroid populations (Figure 2a). Since the dominant factor appears to be the relative proximity of a cell to either the spheroid surface or the necrotic region, rather than absolute distance from the surface *per se*, we have chosen to intercompare results from different spheroid populations by always sorting 10 fractions and grouping on the basis of the fraction *number*. The consistency of the data in Figure 2b argues favourably for this approach.

Combination treatments with CCNU and cisplatin (Figure 3) produced the desired result of more uniform cell killing throughout the spheroid than achievable with equitoxic ex-

**Figure 2** Representative 'toxicity profiles' for 2 h CCNU exposures for V79 spheroids (**a**) and the relative sensitivity of selected cell subpopulations to CCNU (**b**). All data are plotted in a similar manner to Figure 1 with the exception that different spheroid populations were analysed in **a**; in **b** data from 20 independent determinations are included. Note that the toxicity differential is reversed for CCNU relative to cisplatin.

**Figure 3** Curves from representative experiments showing survival of cells sorted from V79 spheroids after exposure to cisplatin, CCNU, or the combination at low (0.25 μg ml$^{-1}$ cisplatin, 0.125 μg ml$^{-1}$ CCNU, **a**) and higher dose intensities (1.0 μg ml$^{-1}$ cisplatin, 0.5 μg ml$^{-1}$ CCNU, **b**). The curves without symbols show the expected survival fraction by fraction for no interaction between the drugs (the product of the cisplatin and CCNU survival values); the much lower observed survivals for both low and high-dose combinations are suggestive of synergy between the agents.

**Figure 4** Comparison of the response of the outermost 10% of cells from V79 spheroids exposed to single-agent cisplatin, single-agent CCNU, or the combination at a 1:1 dose ratio (plotted versus the arithmetic sum of the drug doses). This representation approximates a dose response, showing effect (cytotoxicity, where S = surviving fraction) as a function of dose, indicating that CCNU was the more potent agent, and that the combination exhibited drug interaction (increased slope). From data transformed and plotted in this manner (see Appendix), the combination index for each treatment combination and each cell subpopulation was calculated as shown in Figure 5. For further reference, the dotted lines show the 'envelope of additivity' for isobologram analysis (see text).

posures to either agent alone. Surprisingly, much more cell killing was observed than expected based on the product of surviving fractions for exposures to each drug as a single agent; this was true for both low (Figure 3a) and high intensity (Figure 3b) treatments. Perhaps of most significance, the apparent interaction between the drugs was observed for *all* cell subpopulations within the spheroids.

More rigorous quantisation of the effects of the two-agent treatments is possible using the 'isobologram' analysis (Steel & Peckham, 1979) or the 'combination index' analysis (Chou & Talalay, 1984). Although the former is arguably more rigorous, the latter has a number of practical advantages (particularly for data display) and consequently was adopted for this report (see Appendix).

Unambiguous conclusions from the combination index model require both a reasonable fit of the experimental data to the median effect equation, and parallelism of the dose–response curves for the single agents when the data are fit to that model (Chou & Talalay, 1984). Examples with our data are shown in Figure 4, where the outermost 10% of the cells were analysed after exposure to cisplatin, CCNU, and the 1:1 combination. The increased slope of the latter curve is indicative of nonexclusive interaction of the agents. It is also immediately evident by the combination index analysis criteria that the combination was interacting synergistically, since Figure 4 shows effect (inverse survival) as a function of dose, and the combination treatment data (hexagons) lie well above the cisplatin curve at all doses, and above the CCNU curve at high doses. For intercomparison with the Isobologram analysis, we have also shown the calculated 'envelope of additivity' (terminology as in Steel & Peckham, 1979) based on the survival curves defined for cisplatin and CCNU in Figure 4; with only one exception, the data for the combination treatments are above that region again indicating supra-additivity. Experimental reproducibility can be easily assessed once more, since each data point represents either the mean or individual survival estimate for every dose evaluated in the independent experiments.

Figure 4 also illustrates both the magnitude of the interaction between these drugs and the difficulties in designing experiments to quantify high degrees of synergism. The highest combination dosage shown was 1.75 μg ml$^{-1}$ cisplatin plus 1.75 μg ml$^{-1}$ CCNU; it produced more than 3 logs of cell kill throughout the spheroids. In contrast, even 3.0 μg ml$^{-1}$ cisplatin as a single agent produced only about 1 log of kill (as can be derived from Figure 4 or seen in Figure 1a). One thus requires analytical techniques which can simultaneously resolve small differences at high survival levels (for single-agent 'control' curves), yet provide data for the very potent combination treatments.

Our approach was to determine the median effect relationship for a range of drug combinations for each of the ten cell subpopulations collected from spheroids. From these data (some 330 individual measurements of cytotoxicity for two-drug treatments) the combination index was calculated as a function of administered dose fraction by fraction through the spheroids for several combinations at differing drug dose ratios. The results are shown in Figure 5, where the figures on the right relate CI to drug exposure (for ease of intercomparison, expressed in terms of the amount of cisplatin in the combination) and cellular position within the spheroid. These figures were constructed from 11, 9 and 13 separate treatment doses for the 1:1, 2:1 and 4:1 cisplatin:CCNU drug ratios respectively. In all cases, synergism ($CI < 1.0$) was observed at high drug levels; the net survival produced is shown as a function of position and drug dose in the left panels. Cytotoxicity toward the innermost cells became greater as the relative CCNU dose increased in the combinations, and even modest CCNU levels produced significant synergy within *all* cell subpopulations for high intensity treatments.

We find it interesting that the interaction seen between CCNU and cisplatin in this system is qualitatively reproduced for BCNU and cisplatin, but quantitatively is greatly diminished. The experiment showing the largest 'interaction' we have seen is shown in Figure 6, where the response of cell subpopulations of the V79 spheroids to 1 h treatments with BCNU, cisplatin, or the combination was compared to the expected (independent action) cell kill from the two agents. The observed cytotoxicity of the combination treatments at either low (Figure 6a) or high intensity (Figure 6b) was only slightly greater than the expected response, suggesting that the modest differences in structure between BCNU and CCNU result in small differences in activity as single agents in this system, but produce a considerable difference in combination regimens (compare Figure 6 with Figure 3, where a dose ratio of 2:1 cisplatin:nitrosourea was used in each case, and comparable levels of cytotoxicity were produced).

### Discussion

The data presented in this report indicate that CCNU and cisplatin can be synergistic in V79 spheroids. Perhaps more importantly, the agents, at appropriate concentrations, were synergistic in all cell subpopulations of the spheroids. Our intent in the initial phase of this study was to simply determine whether the complementary cytotoxicity patterns of the two agents throughout the spheroids would be maintained in a multi-agent exposure (i.e. were the microenvironmental factors responsible for differential platinum activity maintained even in a perturbed system?). Clearly, the data indicate that these differences were indeed maintained.

The degree of synergism demonstrated by these two agents was not expected, and probably would have been discovered only by chance had we not been selecting drug combinations based on cell sorting studies of complementary cytotoxicity. Synergy is not specific to spheroids; we have seen similar levels for cisplatin and CCNU treatments of monolayer and suspension cultures of single cells (data not shown). However, the observed synergism in all subpopulations of cells in spheroids provides a compelling argument to consider this drug combination for clinical regimens. 'Non-cross-resistance' of drugs is an established clinical principle for combination chemotherapy; if microenvironmental resistance is included in that definition, then agents like cisplatin and CCNU, which have different normal tissue toxicities as well as different cytotoxicity profiles, emerge as being of potential utility in combination.

**Figure 5** Response surfaces showing cell survival (left panels), or cisplatin/CCNU interaction (right panels), as a function of position in spheroid and drug exposure. For ease of comparison, only the cisplatin dose is shown on the panel scales; note that in the upper panels, 2 $\mu g\ ml^{-1}$ cisplatin corresponds to 2 $\mu g\ ml^{-1}$ cisplatin + 2 $\mu g\ ml^{-1}$ CCNU whereas in the lower panels, a slightly lower total dose of 2 $\mu g\ ml^{-1}$ cisplatin + 0.5 $\mu g\ ml^{-1}$ CCNU was administered. CI approximates the reduction in total dose necessary to achieve a given endpoint, thus indicating a strong interaction between cisplatin and CCNU at higher dose levels. As indicated in the Appendix, CI values at low treatment intensity tend to overestimate antagonism; for clarity, the response surfaces have consequently been constrained to values of 1.0 or less. The top panels were generated for drug doses ranging from 0.25 $\mu g\ ml^{-1}$ cisplatin + 0.25 $\mu g\ ml^{-1}$ CCNU to 1.75 $\mu g\ ml^{-1}$ cisplatin + 1.75 $\mu g\ ml^{-1}$ CCNU; the middle panels for 0.25 $\mu g$ ml cisplatin + 0.125 $\mu g\ ml^{-1}$ CCNU to 2.0 $\mu g\ ml^{-1}$ cisplatin + 1.0 $\mu g\ ml^{-1}$ CCNU, and the lower panels for 0.4 $\mu g$ ml cisplatin + 0.1 $\mu g\ ml^{-1}$ CCNU to 3.2 $\mu g\ ml^{-1}$ cisplatin + 0.8 $\mu g\ ml^{-1}$ CCNU (nine of the 13 doses evaluated were within the 0–2 $\mu g\ ml^{-1}$ cisplatin range shown on the plot).

It must be remembered that the cells differentially resistant to cisplatin in the interior of the spheroid, or those more resistant to CCNU near the exterior of the spheroid, are likely to be different from 'genetically' resistant cells that appear during multiple courses of treatment. This point is of relevance in view of the known $O^6$-methylguanine-DNA methyltransferase resistance phenotype seen in many human cell lines in response to nitrosoureas (the $Mer^+$ phenotype, see Day *et al.*, 1980, 1987). Unlike most human cells, the Chinese hamster cell lines used in these studies are quite sensitive to nitrosoureas. In the clinical situation, as well, it must be noted that the $Mer^+$ or $Mer^-$ phenotype may be of less relevance than whether a relative difference in resistance of tumour and normal cells is present (Day *et al.*, 1987). Preliminary experiments in our laboratory with a human colon carcinoma cell line (WiDR) grown as spheroids have indicated a synergistic interaction between cisplatin and CCNU, despite resistance to CCNU in those cells. One might

**Figure 6** Curves from representative experiments showing survival of cells sorted from V79 spheroids after a 1 h exposure to cisplatin, BCNU, or the combination at low (2.0 μg ml$^{-1}$ cisplatin, 1.0 μg ml$^{-1}$ BCNU, **a**) and high dose intensities (6.0 μg ml$^{-1}$ cisplatin, 3.0 μg ml$^{-1}$ BCNU, **b**). The curves without symbols show the expected survival fraction by fraction for no interaction between the drugs (product of the cisplatin and BCNU survivals); the similarity of the observed and expected survivals for both low and high-dose combinations are suggestive of little interaction between the agents (particularly in relation to Figure 3).

hope, as well, that an analogy between the current demonstration of synergism between cisplatin and CCNU might parallel the recent report (Mulcahy *et al.*, 1988) that Mer$^+$ resistance can be overcome by using the synergistic combination of CCNU and mild hyperthermia.

We do not, at present, have an explanation for the lack (or at least, marked decrease) of synergism between BCNU and cisplatin. This is not, however, the only difference noted between BCNU and CCNU in this system. Another example is the duration of drug exposure; prolonging BCNU exposure beyond 30 min produces little additional cytotoxicity in spheroids. In contrast, CCNU cytotoxicity increases with increasing exposure times for at least the first 12 h of exposure (as does cisplatin cytotoxicity; data not shown). Since our intent in these experiments was to evaluate the synergistic activity of 'simultaneous' drug exposures, we limited the total exposure time to 1 h for the BCNU/cisplatin combinations.

Two additional features should also be noted. The role of hypoxic cells is receiving increased attention in clinical chemotherapy due to potential problems of the microenvironment (Sutherland, 1988), lack of drug delivery (Nederman *et al.*, 1981; Durand, 1989), and perhaps inherent resistance of those noncycling cells (due in part to potential stimulation/overexpression of 'resistance genes' by transient hypoxia, e.g. Rice *et al.*, 1986; Young *et al.*, 1988). One of the more topical methods to combat this feared resistance is the use of 'bioreductive' drugs which are activated in a hypoxic environment and thus preferentially toxic to hypoxic cells (Kennedy *et al.*, 1981). We have evaluated a number of such agents in the spheroids; it is of considerable interest to us that none of these agents (with the possible exception of porfiromycin. a mitomycin C analogue) shows any more preferential activity against the innermost cells of the spheroids than either BCNU or CCNU. This also leads us to speculate that the 'lack' of clinical activity of the nitrosoureas may in fact be linked to their propensity for killing quiescent, non-proliferating cells of solid tumours – activity that may not be easily appreciated in the clinical situation where regression (or regrowth) is typically the only parameter that can be quantified. However, the demonstration of preferential killing of hypoxic/quiescent cells by CCNU, a well established, clinically useful agent may provide an additional opportunity for design of combination chemotherapy protocols, particularly since cell subpopulations differentially sensitive to CCNU may be those resistant to cisplatin.

### Appendix: analysis of interactions

As previously stated, we have used the analytical procedures developed by Chou and Talalay (1984), where survival (S) is related to dose (D) according to the 'median-effect' equation:

$$(1 - S)/S = (D/D_m)^m$$

where m is the sigmoidicity of the curve, and $D_m$ the median dose (which produces 50% survival).

A log transform of the equation simplifies solution for the constants; resulting parallel curves indicate that the treatment agents can be added by dose, and the dose giving survival S is then:

$$D = D_m[(1 - S)/S]^{1/m}$$

The fractional effect ($f_1$) due to drug 1 in a two-drug scheme then varies with its concentration ($C_1$):

$$f_1 = C_1/(C_1 + C_2)$$

Thus, the 'combination index' (CI) can be defined:

$$CI = \frac{(D_{12})\ (f_1)}{D_1} + \frac{(D_{12})\ (f_2)}{D_2} + \frac{(D_{12})\ (f_1)\ (D_{12})\ (f_2)}{(D_1)\ (D_2)}$$

where the last term is required only if the agents are non-exclusive (m is greater for the combined treatment than for either single agent as observed for all data presented here and shown in Figure 4). In essence, the combination index CI is the ratio of the combination dose to the sum of the (isoeffective) single-agent doses; consequently, $CI < 1$ shows potentiation (synergism) and $CI > 1$ indicates antagonism (protection).

The combination index has a number of advantages, and some disadvantages (e.g. Durand & Goldie, 1987). On the positive side, it allows a numeric estimation of the degree of agent interaction, and, as indicated by the last equation above, immediately expresses the degree of interaction as a function of the level of toxicity produced (within the limitation, of course, that the two agents must be administered at a fixed dose ratio). As shown in Figure 5, it is thus possible to simultaneously relate the amount of interaction, the administered drug dose, and cellular position in the spheroid. Functionally, this was performed by generating the median-effect plots fraction by fraction through the spheroid for each drug dose ratio, then interpolating between fractions by using a polynomial fitting routine. From Figures 1a, 2a, 3 and 6 it should be obvious that subpopulations of cells were located at different depths in spheroids of different sizes; we consequently expressed position in terms of the fraction *number* rather than depth.

On the negative side, the combination index analysis requires that the data are adequately described by the median effect equation. While this can generally be achieved over a limited range of doses, there is necessarily some uncertainty introduced by extrapolation beyond the actual ranges of observation. This is perhaps best illustrated by comparison of Figure 5, where antagonism is suggested at low doses, with Figure 3a, which clearly shows strong synergy even for very modest levels of cell killing. The discrepancy, as shown in Figure 4, is that the best-fit line for combinations, if increased in slope, necessarily extrapolates below either single-agent curve at low doses (a concave upward curve is suggested by the actual data). However, this problem is of less significance when considered with the perspective that the combination index analysis produces a quite conservative estimate of synergism: low dose antagonism is *overestimated* by using the linear best-fit, and high dose synergism is, for the same reason, *underestimated*.

This research was supported by MRC grant MA-10238 and USPHS grant CA-37775. The technical assistance of Denise McDougal, Nancy LePard and Sandra Vanderbyl is gratefully acknowledged, as are numerous helpful discussions with Dr James Goldie.

### References

CHOU, T.C. & TALALAY, P. (1984). Quantitative analysis of dose-effect relationships: the combined effects of multiple drugs or enzyme inhibitors. *Adv. Enzyme Regul.*, **22,** 27.

DAY, R.S. III, ZIOLKOWSKI, C.H.J., SCUDIERO, D.A. & 5 others (1980). Defective repair of alkylated DNA by human tumour and SV40-transformed human cell strains. *Nature*, **288,** 724.

DAY, R.S. III, BABICH, M.A., YAROSH, D.B. & SCUDIERO, D.A. (1987). The role of $O^6$-methylguanine in human cell killing, sister-chromatid exchange induction and mutagenesis: a review. *J. Cell Sci. Suppl.*, **6,** 333.

DURAND, R.E. (1976). Cell cycle kinetics in an *in vitro* tumor model. *Cell Tissue Kinet.*, **9,** 403.

DURAND, R.E. (1986*a*). Use of a cell sorter for high precision assays of cell clonogenicity. *Cancer Res.*, **46,** 2775.

DURAND, R.E. (1986*b*). Chemosensitivity testing in V79 spheroids: drug delivery and cellular microenvironment. *J. Natl Cancer Inst.*, **77,** 247.

DURAND, R.E. & GOLDIE, J.H. (1987). Interaction of etoposide and cisplatin in an *in vitro* tumor model. *Cancer Treat. Rep.*, **71,** 673.

DURAND, R.E. (1989). Distribution and activity of antineoplastic drugs in a tumor model. *J. Natl Cancer Inst.*, **81,** 146.

KENNEDY, K.A., TEICHER, B.A., ROCKWELL, S. & SARTORELLI, A.C. (1981). Chemotherapeutic approaches to cell populations of tumors. In *Molecular Actions and Targets for Cancer Chemotherapeutic Agents*, Sartorelli, A.C., Layo, J.S. & Bertino, J.R. (eds), p. 85. Academic Press: New York.

MITCHELL, E.P. & SCHEIN, P.S. (1986). Contributions of nitrosoureas to cancer treatment. *Cancer Treat. Rep.*, **70,** 31.

MULCAHY, R.T., GIPP, J.J. & TANNER, M.A. (1988). Sensitization of nitrosourea-resistant $Mer^+$ human tumor cells to *n*-(2-chloroethyl)-*n'*-cyclohexyl-*n*-nitrosourea by mild (41°C) hyperthermia. *Cancer Res.*, **48,** 1086.

NEDERMAN, T., CARLSSON, J. & MALMQUIST, M. (1981). Penetration of substances into tumor tissue–a methodological study on cellular spheroids. *In Vitro*, **17,** 290.

OLIVE, P.L., LEONARD, J.C. & DURAND, R.E. (1982). Development of tetraploidy in V79 spheroids. *In Vitro*, **18,** 708.

PRESTAYKO, A.W., D'AOUST, J.C., ISSEL, B.F. & CROOKE, S.T. (1979). Cisplatin (*cis*-diamminedichloro-platinum II). *Cancer Treat. Rev.*, **6,** 17.

RICE, G.C., HOY, C. & SCHIMKE, R.T. (1986). Transient hypoxia enhances the frequency of dihydrofolate reductase gene amplification in Chinese hamster ovary cells. *Proc. Natl Acad. Sci. USA*, **83,** 5978.

SCHABEL, F.M. Jr., TRADER, M.W., LASTER, W.R. Jr., CORBETT, T.H. Jr. & GRISWOLD, D.P. Jr. (1979). *Cis*-dichlorodiammine-platinum(I): combination chemotherapy and cross-resistance studies with tumors of mice. *Cancer Treat. Rep.*, **63,** 1459.

STEEL, G.G. & PECKHAM, M.J. (1979). Exploitable mechanisms in combined radiotherapy–chemotherapy: the concept of additivity. *Int. J. Radiat. Oncol. Biol. Phys.*, **5,** 85.

SUTHERLAND, R.M. & DURAND, R.E. (1976). Radiation response of multicell spheroids – an *in vitro* tumor model. *Curr. Top. Radiat. Res. Q.*, **11,** 87.

SUTHERLAND, R.M. (1988). Cell and environment interactions in tumor microregions: the multicell spheroid. *Science*, **240,** 177.

YOUNG, S.D., MARSHALL, R.S. & HILL, R.P. (1988). Hypoxia induces DNA overreplication and enhances metastatic potential of murine tumor cells. *Proc. Natl Acad. Sci. USA*, **85,** 9533.